# Association of Apolipoprotein E With Intracerebral Hemorrhage Risk by Race/Ethnicity: A Meta-analysis

**DOI:** 10.1001/jamaneurol.2018.4519

**Published:** 2019-02-06

**Authors:** Sandro Marini, Katherine Crawford, Andrea Morotti, Myung J. Lee, Alessandro Pezzini, Charles J. Moomaw, Matthew L. Flaherty, Joan Montaner, Jaume Roquer, Jordi Jimenez-Conde, Eva Giralt-Steinhauer, Roberto Elosua, Elisa Cuadrado-Godia, Carolina Soriano-Tarraga, Agnieszka Slowik, Jeremiasz M. Jagiella, Joanna Pera, Andrzej Urbanik, Alexander Pichler, Björn M. Hansen, Jacob L. McCauley, David L. Tirschwell, Magdy Selim, Devin L. Brown, Scott L. Silliman, Bradford B. Worrall, James F. Meschia, Chelsea S. Kidwell, Fernando D. Testai, Steven J. Kittner, Helena Schmidt, Christian Enzinger, Ian J. Deary, Kristiina Rannikmae, Neshika Samarasekera, Rustam Al-Shahi Salman, Catherine L. Sudlow, Catharina J. M. Klijn, Koen M. van Nieuwenhuizen, Israel Fernandez-Cadenas, Pilar Delgado, Bo Norrving, Arne Lindgren, Joshua N. Goldstein, Anand Viswanathan, Steven M. Greenberg, Guido J. Falcone, Alessandro Biffi, Carl D. Langefeld, Daniel Woo, Jonathan Rosand, Christopher D. Anderson

**Affiliations:** 1 Center for Genomic Medicine, Massachusetts General Hospital, Boston; 2 Stroke Unit, IRCCS Mondino Foundation, Pavia, Italy; 3 Department of Neurology, Massachusetts General Hospital, Boston; 4 Department of Clinical and Experimental Sciences, Neurology Clinic, University of Brescia, Brescia, Italy; 5 Department of Neurology and Rehabilitation Medicine, University of Cincinnati College of Medicine, Cincinnati, Ohio; 6 Neurovascular Research Laboratory and Neurovascular Unit, Institut de Recerca, Hospital Vall d’Hebron, Universitat Autonoma de Barcelona, Barcelona, Spain; 7 Institute de Biomedicine of Seville, IBiS/Hospital Universitario Virgen del Rocío/CSIC/University of Seville, Seville, Spain; 8 Department of Neurology, Hospital Universitario Virgen Macarena, Seville, Spain; 9 Department of Neurology, Neurovascular Research Unit, Institut Hospital del Mar d’Investigacions Mèdiques, Universitat Autonoma de Barcelona, Barcelona, Spain; 10 Department of Neurology, Jagiellonian University Medical College, Krakow, Poland; 11 Department of Clinical Sciences Lund, Neurology, Lund University, Lund, Sweden; 12 Department of Neurology and Rehabilitation Medicine, Skåne University Hospital, Lund, Sweden; 13 John P. Hussman Institute for Human Genomics, University of Miami, Miller School of Medicine, Miami; 14 Stroke Center, Harborview Medical Center, University of Washington, Seattle; 15 Department of Neurology, Stroke Division, Beth Israel Deaconess Medical Center, Boston, Massachusetts; 16 Cardiovascular Center, University of Michigan, Ann Arbor; 17 Department of Neurology, University of Florida College of Medicine, Jacksonville; 18 Department of Neurology and Public Health Sciences, University of Virginia Health System, Charlottesville; 19 Department of Neurology, Mayo Clinic, Jacksonville, Florida; 20 Department of Neurology, University of Arizona, Tucson; 21 Department of Neurology and Rehabilitation, University of Illinois College of Medicine, Chicago; 22 Department of Neurology, Baltimore Veterans Administration Medical Center and University of Maryland School of Medicine, Baltimore; 23 Department of Neurology, Medical University of Graz, Graz, Austria; 24 Centre for Cognitive Ageing and Cognitive Epidemiology, University of Edinburgh, Edinburgh, United Kingdom; 25 Centre for Clinical Brain Sciences, University of Edinburgh, Edinburgh, United Kingdom; 26 Centre for Medical Informatics, Usher Institute of Population Health Sciences and Informatics, University of Edinburgh, Edinburgh, United Kingdom; 27 Department of Neurology, Radboud University Medical Centre, Donders Institute for Brain, Cognition and Behaviour, Nijmegen, the Netherlands; 28 Department of Neurology and Neurosurgery, Brain Center Rudolf Magnus, University Medical Center Utrecht, Utrecht, the Netherlands; 29 Stroke Pharmacogenomics and Genetics, Sant Pau Institute of Research, Hospital de la Santa Creu i Sant Pau, Barcelona, Spain; 30 Division of Neurocritical Care and Emergency Neurology, Department of Neurology, Yale University School of Medicine, New Haven, Connecticut; 31 Center for Neuroepidemiology and Clinical Neurological Research, Yale School of Medicine, Yale University, New Haven, Connecticut; 32 Division of Behavioral Neurology, Massachusetts General Hospital, Boston; 33 Center for Public Health Genomics and Department of Biostatistical Sciences, Wake Forest University, Winston-Salem, North Carolina; 34 Program in Medical and Population Genetics, Broad Institute, Cambridge, Massachusetts

## Abstract

**Importance:**

Genetic studies of intracerebral hemorrhage (ICH) have focused mainly on white participants, but genetic risk may vary or could be concealed by differing nongenetic coexposures in nonwhite populations. Transethnic analysis of risk may clarify the role of genetics in ICH risk across populations.

**Objective:**

To evaluate associations between established differences in ICH risk by race/ethnicity and the variability in the risks of apolipoprotein E (*APOE*) ε4 alleles, the most potent genetic risk factor for ICH.

**Design, Setting, and Participants:**

This case-control study of primary ICH meta-analyzed the association of *APOE* allele status on ICH risk, applying a 2-stage clustering approach based on race/ethnicity and stratified by a contributing study. A propensity score analysis was used to model the association of *APOE* with the burden of hypertension across race/ethnic groups. Primary ICH cases and controls were collected from 3 hospital- and population-based studies in the United States and 8 in European sites in the International Stroke Genetic Consortium. Participants were enrolled from January 1, 1999, to December 31, 2017. Participants with secondary causes of ICH were excluded from enrollment. Controls were regionally matched within each participating study.

**Main Outcomes and Measures:**

Clinical variables were systematically obtained from structured interviews within each site. *APOE* genotype was centrally determined for all studies.

**Results:**

In total, 13 124 participants (7153 [54.5%] male with a median [interquartile range] age of 66 [56-76] years) were included. In white participants, *APOE* ε2 (odds ratio [OR], 1.49; 95% CI, 1.24-1.80; *P* < .001) and *APOE* ε4 (OR, 1.51; 95% CI, 1.23-1.85; *P* < .001) were associated with lobar ICH risk; however, within self-identified Hispanic and black participants, no associations were found. After propensity score matching for hypertension burden, *APOE* ε4 was associated with lobar ICH risk among Hispanic (OR, 1.14; 95% CI, 1.03-1.28; *P* = .01) but not in black (OR, 1.02; 95% CI, 0.98-1.07; *P* = .25) participants. *APOE* ε2 and ε4 did not show an association with nonlobar ICH risk in any race/ethnicity.

**Conclusions and Relevance:**

*APOE* ε4 and ε2 alleles appear to affect lobar ICH risk variably by race/ethnicity, associations that are confirmed in white individuals but can be shown in Hispanic individuals only when the excess burden of hypertension is propensity score–matched; further studies are needed to explore the interactions between *APOE* alleles and environmental exposures that vary by race/ethnicity in representative populations at risk for ICH.

## Key Points

**Question:**

Is history of hypertension and apolipoprotein E (*APOE*) associated with intracerebral hemorrhage risk in participants stratified by self-reported race/ethnicity?

**Findings:**

In this case-control study of 13 124 adults, having a copy of *APOE* ε4 alleles increased the risk for lobar intracerebral hemorrhage only in white individuals, but after propensity score matching for hypertension burden, Hispanic individuals showed the same risk of *APOE* ε4.

**Meaning:**

*APOE* ε4 appears to be confirmed as a risk factor for lobar intracerebral hemorrhage in nonwhite populations but is masked by differential hypertension burden in Hispanic individuals; further studies are needed to explore the interactions between *APOE* alleles and environmental exposures.

## Teaser

This case-control study examines whether the risk for intracerebral hemorrhage presented by apolipoprotein E ε4 and ε2 alleles varies by race/ethnicity.

## Introduction

Spontaneous intracerebral hemorrhage (ICH) is the most severe form of stroke. In the United States, 160 000 people experience an ICH each year with a case fatality rate of 54% at 1 year.^1^ The prevalence of ICH has increased 47% between 1990 and 2010,^2^ and ICH risk appears to vary among white, black and Hispanic populations.^3,4,5,6^ Compared with white individuals, young and middle-aged black individuals have an almost 2-fold increased risk for ICH.^3,4^ Similarly, Hispanic individuals have a relative risk increase that ranges from 1.4 for lobar ICH to 3.7 for nonlobar ICH.^5^ Moreover, not only is hypertension prevalence among older adults lower among non-Hispanic white groups (76.3%) than among non-Hispanic black (82.5%) and Hispanic (79.2%) groups, but the risk of ICH in the presence of hypertension increases more than 50% from white to Hispanic groups.^7,8,9^ The associations of genetic and acquired ICH risk factors with these observed risk differences are poorly understood.

Previous studies conducted in predominantly European-ancestry populations have demonstrated that apolipoprotein E (*APOE* [OMIM 107741]) ε2 and ε4 alleles potently increase risk of lobar ICH.^10^ In Alzheimer disease, another disorder associated with *APOE* ε allele status, the degree of risk contributed by *APOE* genotype varies substantially by the ancestry of the population studied. Among non-Hispanic white people, homozygous carriers of *APOE* ε4 exhibit up to a 12-fold higher risk of Alzheimer disease, but this same haplotype exerts little or no risk for black or Hispanic people.^11,12,13^

Understanding how genetic risk factors vary across race/ethnicity may highlight novel underlying disease mechanisms and identify populations who may be particularly responsive to specific prevention strategies, as has previously been shown in treatment response for heart failure by race/ethnicity.^14^ Unfortunately, with individuals of African American and Hispanic ancestry representing less than 4% of all samples in genome-wide association studies, only recently has it become possible to study genetic risk of common disease across representative US populations.^15^

We tested the associations of *APOE* ε alleles with risk of lobar and nonlobar ICH among white, black, and Hispanic individuals, using direct genotyping data supplemented by genome-wide genotyping as available in cases and controls from the International Stroke Genetics Consortium. Because these analyses revealed substantial heterogeneity by race/ethnicity, we further explored the degree to which the differential burden of hypertension across populations is associated with the variability in observed *APOE* risks.

## Methods

### Participating Studies and Data Collection

Case and control participants included in the study were gathered from 3 multicenter studies in the United States and from 8 European sites participating in the International Stroke Genetics Consortium, according to availability of directly ascertained *APOE* ε genotypes and a harmonized local acute case recruitment scheme. These participants were enrolled in the aforementioned studies from January 1, 1999, to December 31, 2017. Institutional review board approval was obtained from all participating centers, and informed consent was obtained from all participants or their legally authorized representative.

The ICH cases from population-based cohorts were not included because of potential imbalances in lethal case recruitment between the 2 sampling approaches.^16^ Studies included the Genetics of Cerebral Hemorrhage with Anticoagulation (GOCHA) study,^17^ the Genetic and Environmental Risk Factors for Hemorrhagic Stroke (GERFHS) study,^18^ the Ethnic/Racial Variations of Intracerebral Hemorrhage (ERICH) study,^19^ the Hospital del Mar and Vall d’Hebron Hospital ICH studies,^20,21^ the Jagiellonian University Hemorrhagic Stroke Study,^22^ the Lund Stroke Register study,^23^ the Edinburgh Stroke Study and LINCHPIN,^24^ the UMC Utrecht ICH study, and the Brescia Stroke Registry.^25^ Because of variable sample sizes from contributing centers, data from European studies were analyzed together for association testing in a meta-analysis (International Stroke Genetics Consortium Europe), as done previously.^26,27^

Participants with secondary causes of ICH were excluded from enrollment. More specific inclusion and exclusion criteria for each of the included studies are reported in eTable 1 in the Supplement. Demographic variables, including self-identified race/ethnicity,^8^ were systematically obtained from structured patient and family member interviews within each site,^19,28^ along with additional covariates.^29^ Computed tomographic images on admission were analyzed at each participating site for classification as lobar (involving predominantly the cortex and underlying white matter) and nonlobar (involving predominately the basal ganglia, periventricular white matter, or internal capsule) according to prespecified criteria.^26,27^ The *APOE* genotype was centrally determined according to standard procedures.^30^ Genomewide data were available for a subgroup of participants. Genetic and bioinformatic analysis followed standardized prespecified quality-control procedures^31^ (eMethods in the Supplement).

### Population Stratification

Fifteen ancestry informative markers were selected from participants with direct or genomewide genotyping and subjected to principal component (PC) analysis in accordance with previously published methods.^32,33,34,35^ The first 4 PCs were included in regression models to adjust for population stratification in this subgroup. This PC analysis was not used to reclassify participants, as self-identified race/ethnicity may capture exposures that transcend genetic ancestry and could help explain the stratification among different populations. A complete description of the genetic analysis, the participants genotyped, and the markers selected is available in eTable 2 in the Supplement.

### Statistical Analysis

Categorical variables were expressed as count (%), and continuous variables were expressed as median (interquartile range [IQR]) or mean (SD), as appropriate. Categorical variables were compared using the 2-tailed χ^2^ test, whereas continuous variables were compared with unpaired Mann-Whitney tests. The threshold for statistical significance was set at *P* = .05.

We tested *APOE* allele association with ICH risk using 3 logistic regression models. Model 1 was adjusted for age, sex, and history of hypertension. Model 2 included variables from model 1 in addition to history of hypercholesterolemia; history of ischemic stroke; warfarin, statin and antiplatelet use; smoking; and alcohol use. Model 3 also included variables from model 1 with the addition of the first 4 principal components derived from ancestry-informative genotypes. *APOE* risk allele status was modeled as 2 variables, ε2 and ε4, coded for allele counts (0, 1, or 2 for each) in an additive model referent to the wildtype ε3 allele.^17^ Analyses were performed in lobar and nonlobar ICH, given the known differences in underlying biology between the 2 ICH locations.^36^ All statistical analyses were performed using Stata, version 13.0 (Stata Corp LLC), and R statistical software (R Foundation for Statistical Computing).

### Transethnic Meta-analysis

We applied a 2-stage clustering approach for meta-analysis, based on race/ethnicity and stratified by study.^37^ Cases and controls in each study were divided into black, white, and Hispanic groups, based on self-identified race/ethnicity. Each race/ethnicity group within each study was allocated to the same cluster and tested using the regression models described above. Individual cluster results were presented graphically by plotting OR estimates on a forest plot to visually assess heterogeneity. The effect sizes obtained were then used for a DerSimonian-Laird random-effects, inverse-weighted nonparametric meta-analysis.^38^ Cochran Q and I^2^ tests were used to quantify heterogeneity.

### Propensity Score Modeling of *APOE* and Hypertension

To address imbalances in the burden of hypertension across ICH populations, and associated imbalances of baseline characteristics among participants with and without hypertension, 2 propensity score (PS) analyses were performed using the nearest neighbor matching method to compare participants of similar underlying hypertensive pathophysiologic burden.^39,40^ The first PS analysis was constructed using history of hypertension and included variables of age, sex, and self-identified race/ethnicity. The second PS analysis, leveraging data only available in the ERICH study, contained the same variables as the first PS analysis, in addition to the number of medications prescribed to treat hypertension as well as systolic and diastolic blood pressure readings at ICH presentation. Propensity score results were used in a logistic regression model for ICH risk identical to model 1, as described. In a sensitivity analysis, the same PS procedure was tested against age (older or younger than 65 years), sex, and hypercholesterolemia to increase the confidence that the PS findings were specific for hypertension.

### Power Calculation

Using empirical data from our analyses, we performed a post hoc calculation of the statistical power to detect an association of *APOE* ε alleles with lobar ICH risk in black and Hispanic participants, commensurate with the effect size detected in white participants. Type I error rate of 0.05, log additive inheritance mode, and 0.01 of population risk were assumed, with analyses performed using Quanto software, version 1.2.4 (University of Southern California).^41^

## Results

In total, 13 124 individuals (47.2% cases) were included from the participating studies. Among this sample, 7153 (54.5%) were male, with a median (IQR) age of 66 (56-76) years and with 8334 white (63.5%), 2272 black (17.3%), 1781 Hispanic (13.6%), and 736 other (5.6%) race/ethnicity (Table). The latter group was excluded from the primary analyses given its low statistical power. Rates of *APOE* ε4 homozygosity in cases were 128 (3.6%) in white, 62 (5.3%) in black, and 22 (1.8%) in Hispanic participants, and the rates of *APOE* ε2 homozygosity in cases were 36 (1.0%) in white, 14 (1.2%) in black, and 5 (0.4%) in Hispanic participants. Among participants, 56.8% (4069 cases and 3379 controls of 13 124 participants) had genomewide or direct genotyping data on ancestry informative markers for PC analysis (eTable 3 in the Supplement). Self-identified race/ethnicity showed overall strong concordance with PC-based ancestry (eFigure in the Supplement). Additional clinical covariates were available for a subset of participants (eTable 4 in the Supplement).

**Table.** Demographic Characteristics and Clinical and *APOE* Allele Frequencies Across Participating Studies

Source	No. (%)			
	ERICH (n = 5017)	GOCHA (n = 2297)	ISGC Europe (n = 3471)	GERFHS (n = 2339)
Male sex	2866 (57.1)	1266 (55.1)	1891 (54.6)	1130 (48.3)
Age, median (IQR)	61 (52-72)	73 (65-80)	70 (61-77)	65 (51-75)
Cases	2880 (57.4)	1322 (57.6)	1281 (36.9)	811 (34.7)
Lobar ICH	882 (30.6)	613 (47.8)	493 (40.2)	316 (39.0)
Nonlobar ICH	1998 (69.4)	670 (52.2)	734 (59.8)	495 (61.0)
Hypertension	3364/4976 (67.6)	1667/2275 (73.3)	1673/2893 (57.8)	1264/2337 (54.1)
Self-reported race/ethnicity				
White	1739 (34.7)	2024 (88.1)	2622 (75.5)	1949 (83.3)
Black	1751 (34.9)	131 (5.7)	NA	390 (16.7)
Hispanic	1527 (30.4)	60 (2.6)	194 (5.6)	NA
Other/missing	NA	82 (3.6)	654 (18.8)	NA
*APOE* ε4 allele count				
0	3553 (70.8)	1664 (71.8)	2789 (80.4)	1664 (71.1)
1	1298 (25.9)	570 (24.8)	637 (18.4)	601 (25.7)
2	166 (3.3)	77 (3.4)	45 (1.3)	74 (3.2)
*APOE* ε2 allele count				
0	4262 (85.0)	1916 (83.4)	3034 (87.4)	1881 (80.4)
1	710 (14.2)	363 (15.8)	413 (11.9)	431(18.4)
2	45 (0.9)	18 (0.8)	24 (0.7)	27 (1.2)

Abbreviations: *APOE*, apolipoprotein E; ERICH, Ethnic/Racial Variations of Intracerebral Hemorrhage; GERFHS, Genetic and Environmental Risk Factors for Hemorrhagic Stroke; GOCHA, Genetics of Cerebral Hemorrhage with Anticoagulation; ICH, intracerebral hemorrhage; IQR, interquartile range; ISGC, International Stroke Genetics Consortium; NA, not applicable.

### Lobar ICH

**Figure 1.**
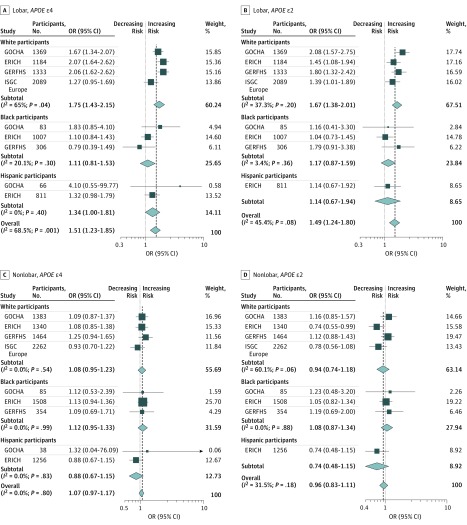
Forest Plots of Meta-analysis of Apolipoprotein E (*APOE)* in Lobar and Nonlobar Intracerebral Hemorrhage Cases and Controls in Model 1, Stratified Across Participating Studies and Race/Ethnicity ERICH indicates Ethnic/Racial Variations of Intracerebral Hemorrhage; GERFHS, Genetic and Environmental Risk Factors for Hemorrhagic Stroke; GOCHA, Genetics of Cerebral Hemorrhage with Anticoagulation; ISGC, International Stroke Genetics Consortium; OR, odds ratio. Weights are from random-effects analysis.

**Figure 2.**
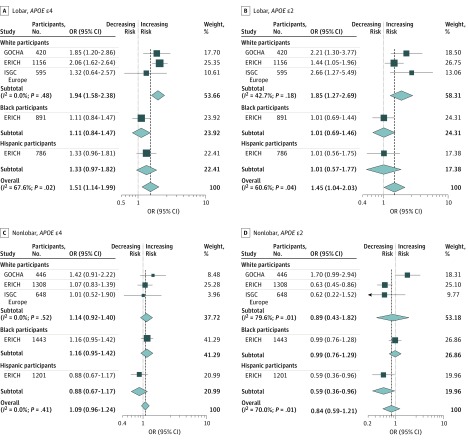
Forest Plots of Meta-analysis of Apolipoprotein E (*APOE)* in Lobar and Nonlobar Intracerebral Hemorrhage Cases and Controls in Model 2, Stratified Across Participating Studies and Race/Ethnicity ERICH indicates Ethnic/Racial Variations of Intracerebral Hemorrhage; GERFHS, Genetic and Environmental Risk Factors for Hemorrhagic Stroke; GOCHA, Genetics of Cerebral Hemorrhage with Anticoagulation; ISGC, International Stroke Genetics Consortium; OR, odds ratio. Weights are from random-effects analysis.

**Figure 3.**
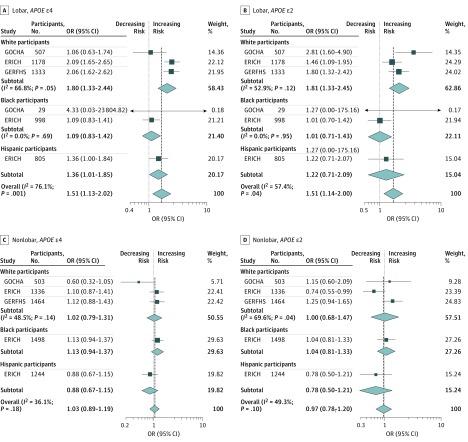
Forest Plots of Meta-analysis of Apolipoprotein E (*APOE)* in Lobar and Nonlobar Intracerebral Hemorrhage Cases and Controls in Model 3, Stratified Across Participating Studies and Race/Ethnicity ERICH indicates Ethnic/Racial Variations of Intracerebral Hemorrhage; GERFHS, Genetic and Environmental Risk Factors for Hemorrhagic Stroke; GOCHA, Genetics of Cerebral Hemorrhage with Anticoagulation; ISGC, International Stroke Genetics Consortium; OR, odds ratio. Weights are from random-effects analysis.

We analyzed 2305 lobar ICH cases from all studies. Model 1 confirmed the previously reported association of *APOE* ε2 (pooled OR, 1.49; 95% CI, 1.24-1.80; *P* < .001) and *APOE* ε4 (pooled OR, 1.51; 95% CI, 1.23-1.85; *P* < .001) with ICH risk; however, within self-identified Hispanic and black groups, no associations were found (Figure 1). Model 2 was used to interrogate the independent association of *APOE* alleles with ICH, controlled for established ICH factors (Figure 2). Here, *APOE* ε2 and ε4 alleles retained an association with lobar ICH. As with model 1, this association was observed in the white group, but not in black or Hispanic groups (for *APOE* ε2 OR, 1.45 [95% CI, 1.04-2.03; *P* = .03]; for *APOE* ε4 OR, 1.51 [95% CI, 1.14-1.99; *P* = .004]). Model 3 considered population stratification (Figure 3). In the white group, both *APOE* ε2 (OR, 1.81; 95% CI, 1.33-2.45; *P* < .001) and *APOE* ε4 (OR, 1.80; 95% CI, 1.33-2.44; *P* < .001) conferred higher risk for lobar ICH. For *APOE* ε4 alone, we found a similar association in the Hispanic group, suggesting that population stratification may have played some role in the lack of ε4 association found in models 1 and 2, particularly for the large and ethnically diverse Hispanic population recruited through the ERICH study. In contrast, for the black population, neither *APOE* ε2 nor *APOE* ε4 conferred a statistically significant risk for lobar ICH after controlling for population structure.

### Nonlobar ICH

We analyzed 3897 nonlobar ICH cases (Figure 1). In model 1, *APOE* ε2 and ε4 did not show an association with nonlobar ICH risk, across any of the self-identified race/ethnicity groups. When comparing nonlobar ICH cases with controls, we found that the *APOE* ε4 *P* values were all *P* > .10. For model 2 and model 3 in nonlobar ICH, again neither *APOE* ε2 nor *APOE* ε4 showed an association with disease risk across all the studies and racial/ethnic groups (Figure 2 and Figure 3).

### Power Calculation (Lobar ICH)

Given the differences in sample sizes between white, black, and Hispanic groups, we performed post hoc power calculations to determine whether the study was powered to detect a comparable *APOE* association in the smaller populations of blacks and Hispanics. Given the frequency of *APOE* ε4 in black participants (ε4 frequency 37.7%), the sample size (assuming an unmatched case-control ratio of 1:1) would provide 99% power to detect an ε4 association similar to the lower bound of the 95% CI seen in white participants (OR, 1.43). Our analyses of *APOE* ε4 association in Hispanic participants were similarly powered at 90%. Furthermore, the *APOE* ε2 frequency in black participants (19.9%) at the reported sample sizes would provide 93.8% power to detect the lower bound of the association seen in white participants (OR, 1.38). For Hispanic participants, given the lower *APOE* ε2 frequency (0.8%), 80% power would be achieved at a slightly higher effect size (OR, 1.60) but still would be below that found in white participants.

### Propensity Score Modeling for Hypertension

**Figure 4.**
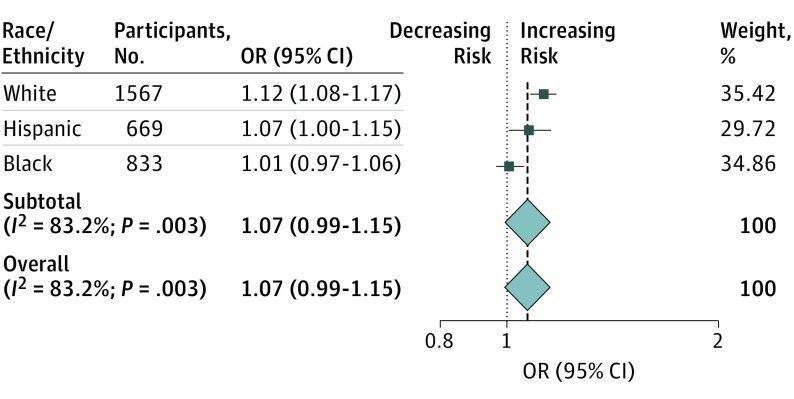
Risk of Apolipoprotein E (*APOE)* ε4 Allele for Lobar Intracerebral Hemorrhage Across Race/Ethnicity After Propensity Score Matching Based on Hypertension Burden Weights are from random-effects analysis.

We used a PS analysis to attempt to isolate the association of *APOE* against the imbalanced burden of hypertension across race/ethnicity. In the first PS, we selected case and control participants with a balanced hypertension burden to form a group comprising individuals of white, black, or Hispanic ancestry. In this matched and homogeneous group, we were able to detect an association of *APOE* ε4 with lobar ICH risk among Hispanic participants (OR, 1.14; 95% CI, 1.03-1.28; *P* = .01) but not in black participants (OR, 1.02; 95% CI, 0.98-1.07; *P* = .25). Results were confirmed in the second PS analysis performed only in the ERICH data set, which included hypertension diagnosis as well as additional hypertension severity variables, including number of medications used to treat hypertension and systolic and diastolic blood pressure readings (Figure 4).

## Discussion

Although *APOE* associations with ICH risk have been characterized in multiple previous studies and meta-analyses for populations with European, and more recently Asian, ancestries, there have been fewer opportunities for examination of US minority populations at disproportionate risk for ICH. Supplemented by data from the ERICH study, we are now able to confirm variability in associations between *APOE* ε genotypes and lobar ICH risk across white, black, and Hispanic groups and to explore the degree to which differences in genetic risk are attributable to comorbid exposures. Our results demonstrate an association of *APOE* ε4 and ε2 alleles with lobar ICH led primarily by white individuals and confirmed by additional models adjusting for known covariates.^29^ When the association of hypertension is propensity matched across race/ethnicity, *APOE* ε4 emerges as a risk factor for lobar ICH among self-identified Hispanic individuals.

These results highlight the challenges of generalizing genetic risk factors across ancestries, where nongenetic exposures are known to vary by race/ethnicity. In Alzheimer disease, the relative risks for Hispanic or black individuals associated with an *APOE* ε 4 allele become progressively weaker or disappear entirely in comparison with white individuals.^42,43,44,45,46^ In ICH, *APOE* ε alleles have already been shown to exert higher risks in East Asian individuals when compared with those of European ancestry.^47^ Although recent analyses by Sawyer et al^48^ demonstrate the association of hypertension and *APOE* ε allele status with ICH risk across race/ethnicity specific to the ERICH study, the present analysis has the advantage of a larger sample size via formal transethnic meta-analysis as well as a PS matching approach that helps to illustrate the potential mechanisms underlying the observed variability of *APOE* ε alleles on lobar ICH risk across populations.

The observed differences in association between *APOE* ε alleles and lobar ICH risk do not provide direct evidence that the biological risks of the *APOE* gene or associations with underlying cerebral amyloid angiopathy (CAA, a major cause of lobar ICH) are necessarily different across racial/ethnic boundaries. It would seem more likely that genetic and/or environmental risk exposures covarying with race/ethnicity exert a role in modifying or mitigating the underlying *APOE* genetic risks. The PS analysis supports this conjecture, demonstrating that hypertension, the most important known risk factor for ICH, may simply obscure the *APOE* risks that may be common across ancestries. Aside from variation in environmental risk exposures, variants in a modifier gene (or genes) that differ across populations may alter the biological risk of *APOE* and consequently vary ICH risk, as has been hypothesized for Alzheimer disease.^49,50^ Furthermore, genetic variants that are racially stratified and not associated with *APOE* may directly modify the risk of ICH. This hypothesis represents an alternative explanation for why the PS matching for hypertension only partially remediated the association of *APOE* ε4 with ICH risk in Hispanic participants and had little to no association in black participants. *APOE* interaction studies and transethnic genome-wide association studies for ICH will likely provide insights on these hypotheses. Similarly, analyses of CAA in nonwhite populations will also clarify the association of race/ethnicity with this pathologic pathway.

In this study, we have not attempted to stratify subsets of participants by probable CAA status using magnetic resonance imaging data as has been done in previous meta-analyses. Most studies linking lobar hemorrhage locations to the pathologic diagnosis of CAA were performed in largely white populations.^51^ As such, widely accepted criteria for classifying probable and possible CAA using hemorrhage location and microbleed counts have not been validated in nonwhite populations.^52^ Validating CAA burdens across multiethnic populations will require concomitant neuroimaging and/or tissue pathologic data in genotyped individuals of many races/ethnicities to ensure patients are not misassigned.

Previously demonstrated associations between *APOE* ε4 and nonlobar ICH risk, also seen in nonlobar ICH recurrence,^17,53^ were not replicated in this study. Potential explanations include a higher rate of participants affected by hypertension and an overall younger age of participants in this study. These factors may reflect the associations of environmental or non-*APOE* genetic exposures in younger populations with nonlobar ICH in particular. Demographic heterogeneity is also higher in this study, and the reduced availability of covariates such as steady state lipid levels^53^ for risk modeling may have affected this finding. In addition, a previous meta-analysis of *APOE* risks in ICH also did not show the association between *APOE* ε4 and nonlobar ICH in black individuals, a finding supported by the present analyses.^17^ Future studies in larger data sets with well-phenotyped cases are needed to further elucidate the potential role of *APOE* ε4 in nonlobar ICH.

### Strengths and Limitations

The targeted enrollment of Hispanic and black individuals through the ERICH study, lacking in previous reports,^17,54^ is an important strength of the present study. The high number of nonwhite individuals enrolled permits well-powered analyses in these populations and promotes confidence that the lack of observed associations is not a false acceptance of the null hypothesis, as supported by our post hoc power calculations.

Some limitations of the study should be acknowledged. Diagnoses of comorbidities were based on self-identified attestation and therefore affected by patient or caregiver awareness. This concern is present in both cases and controls, however, and internal consistency between diagnoses and prescribed medications helps to limit this potential source of bias. Furthermore, the propensity score was based on variables that only partially captured the complex phenotype represented by hypertension. However, this lack of information content is likely to bias our score results toward the null; we expect that a more precise index of hypertension burden would have increased our ability to normalize this phenotype across race/ethnicity and to demonstrate even more homogeneous *APOE* ε4 risks. Finally, genomewide genotypes for the ERICH study participants are not currently available, preventing us from determining whether additional genetic exposures modify the association of *APOE* with ICH risk across race/ethnicity.

## Conclusions

In this meta-analysis, *APOE* ε2 and ε4 alleles remain genetic risk factors for lobar ICH, but these results are largely driven by the associations in white individuals. However, the results support a biological risk of *APOE* ε4 alleles that seems to transcend ancestral backgrounds,^55^ albeit with varying effect because of the presence of racial/ethnic disparities across associated risk factors. As availability of genetic data on US minority populations continues to increase, it is hoped that improved modeling of covarying genetic and nongenetic exposures in these populations will provide new insights into treatment and prevention strategies in ICH that maximize the potential advantages for all.

## Notes

Supplement. **eMethods.** **eTable 1.** ICH Case Inclusion and Exclusion Criteria by Recruitment Site **eTable 2.** Ancestry Informative Markers Selected **eTable 3.** Comparison Between With Subjects With and Without Ancestry Informative Markers Available for Population Stratification Analysis **eTable 4.** Frequencies of Additional Diseases and Risk Factors Among Cases and Controls of the Available Data **eFigure.** Distribution of Subjects in Principal Component (PA) Space and the Relation with Self-identified Race and Ethnicity (Ellipses Cluster 95% of PC Analysis Data)
