# Supplementary material for: Association of Apolipoprotein E With Intracerebral Hemorrhage Risk by Race/Ethnicity: A Meta-analysis
Source: JAMA Neurol. 2019 Feb 6;76(4):480–91. doi: 10.1001/jamaneurol.2018.4519 (PMC6459133; doi:10.1001/jamaneurol.2018.4519)
Supplement: Supplement. — eMethods. eTable 1. ICH Case Inclusion and Exclusion Criteria by Recruitment Site eTable 2. Ancestry Informative Markers Selected eTable 3. Comparison Between With Subjects With and Without Ancestry Informative Markers Available for Population Stratification Analysis eTable 4. Frequencies of Additional Diseases and Risk Factors Among Cases and Controls of the Available Data eFigure. Distribution of Subjects in Principal Component (PA) Space and the Relation with Self-identified Race and Ethnicity (Ellipses Cluster 95% of PC Analysis Data) [file jamaneurol-76-480-s001.pdf]

Supplementary Online Content

Marini S, Crawford K, Morotti A, et al; the International Stroke Genetics Consortium. Association of apolipoprotein E with intracerebral hemorrhage risk by race/ethnicity: a meta-analysis. *JAMA Neurol.* Published online February 6, 2019. doi:10.1001/jamaneurol.2018.4519

eMethods

eTable 1. ICH Case Inclusion and Exclusion Criteria by Recruitment Site

eTable 2. Ancestry Informative Markers Selected

eTable 3. Comparison Between With Subjects With and Without Ancestry Informative Markers Available for Population Stratification Analysis

eTable 4. Frequencies of Additional Diseases and Risk Factors Among Cases and Controls of the Available Data

eFigure. Distribution of Subjects in Principal Component (PA) Space and the Relation with Self-identified Race and Ethnicity (Ellipses Cluster 95% of PC Analysis Data)

This supplementary material has been provided by the authors to give readers additional information about their work.

© 2019 Marini S et al. *JAMA Neurology*.

Apolipoprotein E and Intracerebral Hemorrhage: A Trans-Ethnic Meta-Analysis

Sandro Marini[1], MD; Katherine Crawford[1], BS; Andrea Morotti[2], MD;
Myung J. Lee[3], BA; Alessandro Pezzini[4], MD; Charles J. Moomaw[5], PhD;
Matthew L. Flaherty[5], MD; Joan Montaner[6,7,8], MD, PhD; Jaume Roquer[9], MD, PhD;
Jordi Jimenez-Conde[9], MD, PhD; Eva Giralt-Steinhauer[9], MD, PhD;
Roberto Elosua[9], MD, PhD; Elisa Cuadrado-Godia[9], MD, PhD;
Carolina Soriano-Tarraga[9], PhD, BSc; Agnieszka Slowik[10], MD, PhD;
Jeremiasz M. Jagiella[10], MD, PhD; Joanna Pera[10], MD; Andrzej Urbanik[10], MD, PhD;
Alexander Pichler[10], MD; Björn M. Hansen[11], MD; Jacob L. McCauley[12], PhD;
David L. Tirschwell[13], MD, MSc; Magdy Selim[14], MD, PhD;
Devin L. Brown[15], MD, MS; Scott L. Silliman[16], MD; Bradford B. Worrall[17], MD, MSc;
James F. Meschia[18], MD; Chelsea S. Kidwell[19], MD; Fernando D. Testai[20], MD;
Steven J. Kittner[21], MD, MPH; Helena Schmidt[22], MD; Christian Enzinger[22], MD;
Ian J. Deary[23], FBA, FRSE, FMedSci; Kristiina Rannikmae[24], MD, PhD;
Neshika Samarasekera[24], PhD, MRCP; Rustam Al-Shahi Salman[24], MA, PhD, FRCP;
Catherine L. Sudlow[24], BMBCh, MSc, DPhil, FRCPE;
Catharina J.M. Klijn[26], MD, PhD; Koen M. van Nieuwenhuizen[26], MD;
Israel Fernandez-Cadenas[9], PhD; Pilar Delgado[6], MD, PhD; Bo Norrving[11], MD;
Arne Lindgren[11], MD; Joshua N. Goldstein[3], MD, PhD; Anand Viswanathan[3], MD, PhD;

© 2019 Marini S et al. *JAMA Neurology*.

Steven M. Greenberg[3], MD, PhD; Guido J. Falcone[27], MD, ScD, MPH;
Alessandro Biffi[28], MD; Carl D. Langefeld[29], PhD; Daniel Woo[5], MD;
Jonathan Rosand[1,3,30], MD, MSc and Christopher D. Anderson[1,3,30], MD, MMSc
on behalf of the International Stroke Genetics Consortium

1 Center for Genomic Medicine, Massachusetts General Hospital, Boston, MA, USA
2 Stroke Unit, IRCCS Mondino Foundation, Pavia, Italy
3 Department of Neurology, Massachusetts General Hospital, Boston, MA, USA
4 Department of Clinical and Experimental Sciences, Neurology Clinic, University of Brescia, Brescia, Italy
5 Department of Neurology and Rehabilitation Medicine, University of Cincinnati College of Medicine, Cincinnati, OH, USA
6 Neurovascular Research Laboratory and Neurovascular Unit, Institut de Recerca, Hospital Vall d'Hebron, Universitat Autonoma de Barcelona, Barcelona, Spain
7 Institute de Biomedicine of Seville, IBiS/Hospital Universitario Virgen del Rocío/CSIC/University of Seville, Seville, Spain;
8 Department of Neurology, Hospital Universitario Virgen Macarena, Seville, Spain
9 Department of Neurology, Neurovascular Research Unit, Institut Hospital del Mar d'Investigacions Mèdiques, Universitat Autonoma de Barcelona, Barcelona, Spain
10 Department of Neurology, Jagiellonian University Medical College, Krakow, Poland
11 Department of Clinical Sciences Lund, Neurology, Lund University, Lund, Sweden and Department of Neurology and Rehabilitation Medicine, Skåne University Hospital, Lund, Sweden
12 John P. Hussman Institute for Human Genomics, University of Miami, Miller School of Medicine, Miami, FL 33136, USA
13 Stroke Center, Harborview Medical Center, University of Washington, Seattle, WA, USA
14 Department of Neurology, Stroke Division, Beth Israel Deaconess Medical Center, Boston, MA, USA
15 Cardiovascular Center, University of Michigan, Ann Arbor, MI, USA
16 Department of Neurology, University of Florida College of Medicine, Jacksonville, FL, USA
17 Department of Neurology and Public Health Sciences, University of Virginia Health System, Charlottesville, VA, USA
18 Department of Neurology, Mayo Clinic, Jacksonville, FL, USA
19 Department of Neurology, University of Arizona, Tucson, AZ, USA
20 Department of Neurology and Rehabilitation, University of Illinois College of Medicine, Chicago, IL, US
21 Department of Neurology, Baltimore Veterans Administration Medical Center and University of Maryland School of Medicine, Baltimore, MD, USA
22 Department of Neurology, Medical University of Graz, Graz, Austria
23 Centre for Cognitive Ageing and Cognitive Epidemiology, University of Edinburgh, Edinburgh, United Kingdom
24 Centre for Medical Informatics, Usher Institute of Population Health Sciences and Informatics, University of Edinburgh, United Kingdom
25 Centre for Clinical Brain Sciences, University of Edinburgh, United Kingdom
26 Department of Neurology, Radboud University Medical Centre; Donders Institute for Brain, Cognition and Behaviour, Nijmegen, and Department of Neurology and Neurosurgery, Brain Center Rudolf Magnus, University Medical Center Utrecht, Utrecht, the Netherlands
27 Division of Neurocritical Care and Emergency Neurology, Department of Neurology, Yale University School of Medicine, New Haven, Connecticut, USA and Center for Neuroepidemiology and Clinical Neurological Research, Yale School of Medicine, Yale University, New Haven, CT, USA
28 Division of Behavioral Neurology, Massachusetts General Hospital, Boston, MA, USA
29 Center for Public Health Genomics and Department of Biostatistical Sciences, Wake Forest University, Winston-Salem, NC, USA
30 Program in Medical and Population Genetics, Broad Institute, Cambridge, MA, USA

© 2019 Marini S et al. *JAMA Neurology*.

Correspondence:
Christopher D. Anderson MD, MMSc
185 Cambridge Street CPZN 6818
Boston, MA 02114
Phone: +1-617-726-4369
e-mail: cdanderson@partners.org

eMethods

Participating Studies

Case and control subjects included in the study were gathered from 3 multicenter studies in the US and from 8 distinct European sites participating in the ISGC, based on availability of directly ascertained APOE ε genotypes. US studies included The Genetics of Cerebral Hemorrhage with Anticoagulation (GOCHA) study[1], the Genetic and Environmental Risk Factors for Hemorrhagic Stroke (GERFHS) study[2], the Ethnic/Racial Variations of Intracerebral Hemorrhage (ERICH) study[3]. European studies included The Hospital del Mar and Vall d'Hebron Hospital ICH studies [4,5] in Barcelona, Spain, the Jagiellonian University Hemorrhagic Stroke Study[6] in Krakow, Poland, the Lund Stroke Register study[7] in Lund, Sweden, the Edinburgh Stroke Study and LINCHPIN[8] study in Scotland, UK, the University Medical Center (UMC) Utrecht ICH study, and the Brescia Stroke Registry[9]. Because of variable sample sizes from contributing centers, data from the European studies (ISGC Europe) were analyzed together for association testing in meta-analysis, as done previously[10,11]. We analyzed primary ICH cases, CT or MRI confirmed, aged >18 years (GOCHA enrolled subjects aged > 50 years). ICH cases where there was evidence of secondary cause (such as trauma, tumor, hemorrhagic transformation of ischemic stroke, or vascular malformation) were excluded. More specific inclusion and exclusion criteria for each of the included studies are reported in eTable 1.

Phenotypic and Genetic variables

© 2019 Marini S et al. *JAMA Neurology*.

Demographic variables, including self-identified race and ethnicity (according to the recommendation of the Standards for Maintaining, Collecting, and Presenting Federal Data on Race and Ethnicity[12]) were systematically obtained from structured patient and family member interview within each site[3,13]. Additional covariates previously associated with ICH[14] risk were also collected: medical history of hypertension, previous stroke, hypercholesterolemia, warfarin, antiplatelet and statin use, smoking history, and alcohol exposure (more than twice per week)[15].Given the heterogeneity among the studies, other variables were not available. Number of medications used to treat hypertension (taken in the 2 weeks before ICH onset or before the interview, for cases and controls respectively) and systolic and diastolic blood pressure readings (measured on enrollment with index ICH for cases, and at the time of interview with controls) were also available for ERICH participants. CT images on admission were analyzed at each participating site for hematoma classification as lobar (involving predominantly the cortex and underlying white matter), and non-lobar (involving predominately the basal ganglia, periventricular white matter, or internal capsule), following prespecified criteria[11].

APOE genotype was determined as part of an ongoing GWAS of ICH. Taqman (Applied Biosystems, Foster City, CA) and iPLEX (Sequenom, San Diego, CA) methodologies were adopted at the University of Miami and Massachusetts General Hospital genotyping centers, respectively for all the sample. The genotypes obtained for rs429358 (C/T) and rs7412 (C/T) were used to define the three standard human APOE ε haplotypes of ε2, ε3, and ε4[16]. Genome-wide data were available for a subgroup of subjects, from Affymetrix 6.0 in GERFHS, and Illumina HumanHap610-Quad in GOCHA and ISGC Europe. IRB approval was obtained at all participating centers, and informed consent was obtained from all participants or their legally authorized representative.

Population Stratification

Fifteen ancestry informative markers were selected among the polymorphisms directly genotyped in ERICH [17] (eTable 2). These markers, distributed across the genome, showed difference in allele frequency (δ) of at least 0.5 between any two of the recruited ancestral populations (African American, Hispanic American, non-Hispanic European). GWAS data for GOCHA, GERFHS, ISGC Europe samples were analyzed as previously described[10]. Briefly

© 2019 Marini S et al. *JAMA Neurology*.

Standardized prespecified quality control procedures[18], imputation via IMPUTE2 v.2.2[19] and 1000 Genomes integrated reference panels (Phase I interim release in NCBI build 37), and post imputation filtering (MAF <0.01, IMPUTE2 information score <0.7) were implemented. Samples with < 90% call rates, or SNPs with call rates <95% or deviation from Hardy-Weinberg equilibrium at P-value $< 2.5 \times 10^{-4}$ were excluded. Based on these 15 selected markers, principal component analysis was implemented in GOCHA, GERFHS, ISGC Europe and ERICH subjects in accordance with previously published methods[20–22]. The first four principal components were included in regression models to adjust for population stratification. We have previously shown the reliability of this approach in defining ancestries of populations[17]. This PC analysis was not used to reclassify participants, as self-identified race/ethnicity may capture exposures that transcend genetic ancestry and could contribute to explain the stratification among different populations.

© 2019 Marini S et al. *JAMA Neurology*.

eTable 1. ICH Case Inclusion and Exclusion Criteria by Recruitment Site

Study	Inclusion Criteria	Exclusion Criteria	Control Recruitment
Brescia Stroke Registry. (University of Brescia, Brescia, Italy)	(Hospital-based, prospective, +18 y/o)<br>• Acute hospitalization for ICH<br>• CT or MRI confirmation of ICH<br>• Age > 18	• Head trauma<br>• Brain tumor<br>• Ischemic stroke<br>• Vascular malformation<br>• Other cause of secondary ICH	• Regionally matched, hospital and ambulatory clinics
UMC Utrecht ICH Study (University Medical Center Utrecht, Utrecht, The Netherlands)	(Hospital-based, prospective, +18 y/o)<br>• Acute hospitalization for ICH<br>• CT confirmation of ICH<br>• Age > 18	• Head trauma<br>• Brain tumor<br>• Ischemic stroke<br>• Vascular malformation<br>• Other cause of secondary ICH present on admission or in follow-up	• Regionally matched, blood donor population
Edinburgh – ESS (Western General Hospital, Edinburgh, Scotland, UK)	(Inpatient and outpatient hospital-based, prospective, +55 y/o)<br>• Acute hospitalization for ICH<br>• CT or MRI confirmation of ICH<br>• Age > 55	• Head trauma<br>• Brain tumor<br>• Ischemic stroke<br>• Vascular malformation<br>• Presentation > 1 week from ICH<br>• Antecedent drug use<br>• Primary coagulopathy	• N/A
Edinburgh – LINCHPIN (Western General Hospital, Royal Infirmary of Edinburgh, St. John's Hospital at Howden, West Lothian, Scotland, UK)	(Community-based in areas served by NHS Lothian Health Board, prospective with hot-pursuit and retrospective augmentation, +16 y/o)<br>• Symptomatic ICH (acute or chronic)	• Head trauma<br>• Brain tumor<br>• Ischemic stroke with hemorrhagic transformation<br>• Vascular malformation<br>• Other cause of secondary ICH	• N/A

© 2019 Marini S et al. *JAMA Neurology*.

	• CT or MRI confirmation of acute or chronic ICH<br>• Age > 16<br>• Resident in area served by NHS Lothian Health Board at time of ICH		
ERICH<br>(19 centers in USA, based at University of Cincinnati)	(Hospital-based, prospective with hot-pursuit, +18 y/o)<br><br>• Acute hospitalization for ICH<br>• CT or MRI confirmation of ICH<br>• Age > 18	• Head trauma<br>• Brain tumor<br>• Ischemic stroke<br>• Vascular malformation<br>• Other cause of secondary ICH	• Regionally matched, random-digit-dialing
GOCHA<br>(6 centers in USA, based at Massachusetts General Hospital)	(Hospital-based, prospective, +55 y/o)<br><br>• Acute hospitalization for ICH<br>• CT or MRI confirmation of ICH<br>• Age > 55	• Head trauma<br>• Brain tumor<br>• Ischemic stroke<br>• Vascular malformation<br>• Other cause of secondary ICH	• Regionally matched, ambulatory clinics
GERFHS<br>(16 centers in the Greater Cincinnati/Northern Kentucky region of USA, based at University of Cincinnati)	(Hospital-based, prospective, +18 y/o)<br><br>• Acute hospitalization for ICH<br>• CT or MRI confirmation of ICH<br>• Age > 18	• Head trauma<br>• Brain tumor<br>• Ischemic stroke<br>• Vascular malformation<br>• Other cause of secondary ICH	• Regionally matched, random-digit-dialing
ISGC Europe ICH studies (Hospital del Mar, Vall d'Hebron Hospital, Jagiellonian University, Lund University)	(Hospital-based, prospective, +18 y/o)<br><br>• Acute hospitalization for ICH<br>• CT or MRI confirmation of ICH<br>• Age > 18	• Head trauma<br>• Brain tumor<br>• Ischemic stroke<br>• Vascular malformation<br>• Other cause of secondary ICH	• Regionally matched, hospital and ambulatory clinics
* Lothian Birth Cohort 1936			• Community population born in 1936 who took Scottish Mental

© 2019 Marini S et al. *JAMA Neurology*.

(All centers serving the Lothian Area of Scotland)			Survey in 1947, living in Lothian, Scotland, UK

* This study was used to recruit controls for the Edinburgh – ESS and Edinburgh – LINCHPIN cases

© 2019 Marini S et al. *JAMA Neurology*.

eTable 2. Ancestry Informative Markers Selected

AIM	Chromosome	position (GRCh37.p13)	A1	A2	Allele frequencies		
					AFR	AMR	EUR
rs7581299	2	179900720	T	C	0.749	0.660	0.370
rs11725412	4	38277754	A	G	0.215	0.464	0.063
rs12640848	4	71506412	A	G	0.967	0.660	0.331
rs1423099	5	75427518	T	C	0.930	0.568	0.264
rs4463276	6	145055331	A	G	0.088	0.376	0.771
rs13259288	8	4483059	T	G	0.822	0.478	0.764
rs12679427	8	72242674	T	C	0.437	0.424	0.724
rs10962599	9	16795286	T	C	0.977	0.651	0.274
rs10840311	11	9854857	T	C	0.726	0.601	0.352
rs3019657	11	134511647	A	G	0.696	0.496	0.871
rs550338	12	24512037	A	G	0.697	0.460	0.764
rs200354	14	99375321	T	G	0.336	0.476	0.829
rs12913832	15	28365618	A	G	0.972	0.798	0.364
rs2216594	19	33533258	A	G	0.048	0.347	0.676
rs801712	22	47090243	C	G	0.251	0.477	0.795

AIM: ancestry informative marker; A1 and A2: allele frequency; AFR: Afro-American; AMR: Mixed American; EUR: European

© 2019 Marini S et al. *JAMA Neurology*.

eTable 3. Comparison Between With Subjects With and Without Ancestry Informative Markers Available for Population Stratification Analysis

	GENETIC DATA for population stratification		
	**available (n 7452)**	**not available (n 5672)**	p value
Male sex, n (%)	4140 (55.6%)	3013 (53.1%)	**0.003**
Age, median (IQR)	66 (54-76)	68 (58-77)	**<0.001**
Cases, n (%)	4069 (54.6)	2225 (39.2)	**<0.001**
ICH location			**<0.001**
Lobar ICH, n (%)	1408/6202 (34.7)	897/6202 (41.9)	
Non-lobar ICH, n (%)	2654/6202 (65.3)	1243/6202 (58.1)	
Hypertension, n (%)	4609/12481 (67.2)	3359/12481 (59.7)	
Self-reported race/ethnicity, n (%)			**<0.001**
white	3594/12449 (52.8)	4971/12449 (88.2)	
blacks	1730/12449 (25.4)	550/12449 (9.8)	
Hispanics	1486/12449 (21.8)	118/12449 (2.1)	
APOE ε4 allele count, n (%)			**<0.001**
	5310 (71.3)	4346 (76.6)	
	1909 (25.6)	1197 (21.1)	
	233 (3.1)	129 (2.3)	
APOE ε2 allele count, n (%)			0.53
	6251 (83.9)	4842 (85.4)	
	1137 (15.3)	780 (13.8)	
	64 (0.9)	50 (0.9)	

© 2019 Marini S et al. *JAMA Neurology*.

eTable 4. Frequencies of Additional Diseases and Risk Factors Among Cases and Controls of the Available Data

	**Controls**	**Cases**	**p value***
Smoking, n (%)	2084 (36.2)	1959 (35.3)	0.170
Alcohol use, n (%)	2414 (47.0)	2087 (41.4)	< 0.001
Previous CV ischemic event, n (%)	371 (7.7)	636 (12.6)	< 0.001
Hypercholesterolemia, n (%)	2748 (46.3)	2315 (40.1)	< 0.001
Statin use, n (%)	1209 (26.6)	1220 (22.7)	< 0.001
Antiplatelet use, n (%)	1186 (35.5)	1655 (32.7)	0.005
Warfarin use, n (%)	404 (6.5)	793 (13.0)	< 0.001
Hypertension, n (%)	3404 (53.0)	4564 (75.3)	< 0.001
Male sex, n (%)	3590 (52.6)	3562 (56.6)	< 0.001
Age, median (IQR)	66 (56-75)	67 (56 -78)	< 0.001

* $\chi 2$ test and Mann Whitney u test; CV: cerebrovascular

© 2019 Marini S et al. *JAMA Neurology*.

eFigure. Distribution of Subjects in Principal Component (PA) Space and the Relation with Self-identified Race and Ethnicity (Ellipses Cluster 95% of PC Analysis Data)

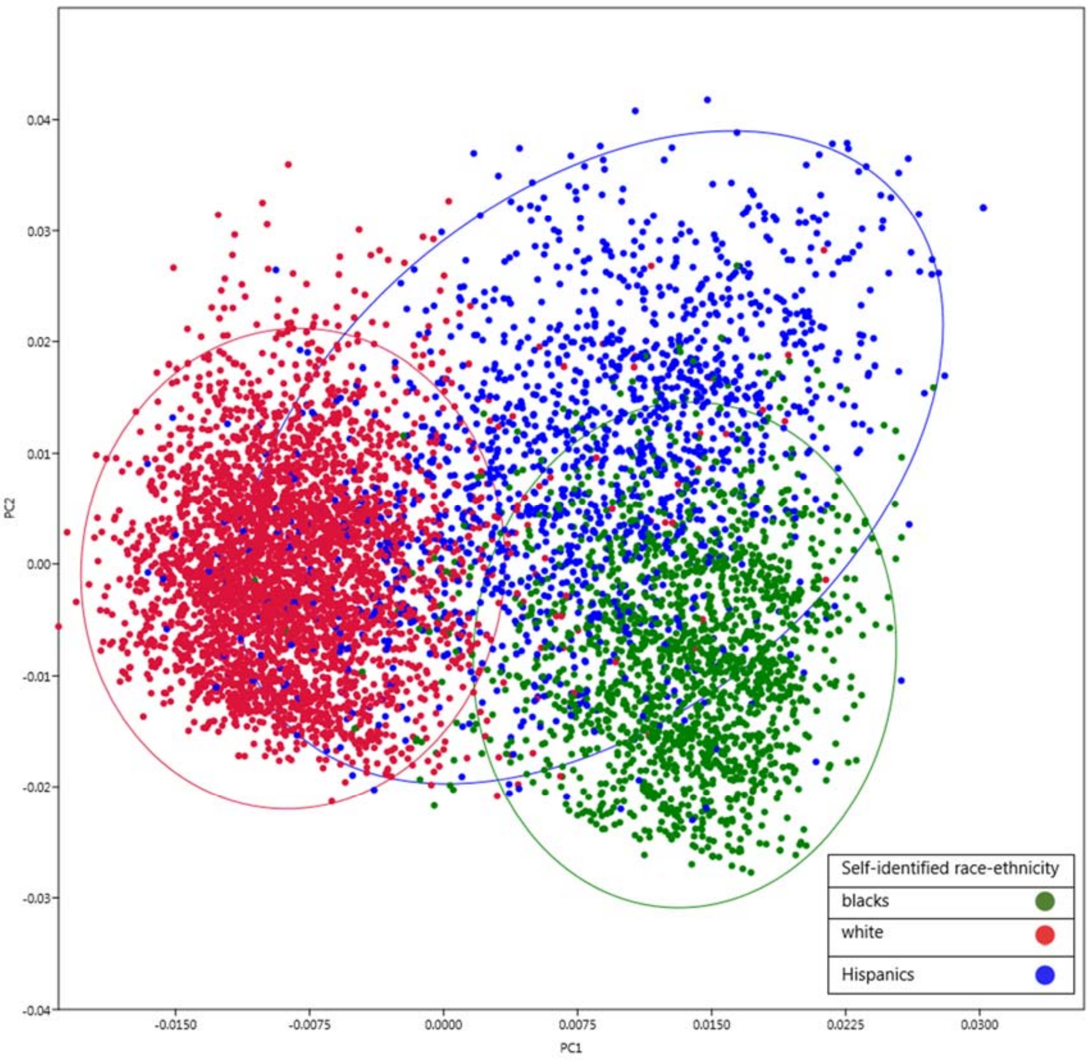

		Ellipses clusters (cluster of 95% data based on PC space)					
		white	Hispanics	blacks	Hispanics - blacks	Hispanics- white	blacks - white
Self-identified race-ethnicity	blacks	-	41	-		30	-
	white	-	38	3	43	-	-
	Hispanics	26	-	19	-	-	-

© 2019 Marini S et al. *JAMA Neurology*.

REFERENCES:

1. Biffi A, Sonni A, Anderson CD, et al. Variants at APOE influence risk of deep and lobar intracerebral hemorrhage. Ann. Neurol. 2010;68(6):934–943.

2. Woo D, Sauerbeck LR, Kissela BM, et al. Genetic and environmental risk factors for intracerebral hemorrhage: preliminary results of a population-based study. [Internet]. Stroke. 2002;33(5):1190–1195.Available from: http://eutils.ncbi.nlm.nih.gov/entrez/eutils/elink.fcgi?dbfrom=pubmed&id=11988589&retmode=ref&cmd=prlinks%5Cnpapers2://publication/uuid/F4CCED2B-2029-4760-BD5C-E16B7C9F166E

3. Woo D, Rosand J, Kidwell C, et al. The ethnic/racial variations of intracerebral hemorrhage (ERICH) study protocol. Stroke 2013;44(10):1–7.

4. Gomis M, Ois A, Rodríguez-Campello A, et al. Outcome of intracerebral haemorrhage patients pre-treated with statins. Eur. J. Neurol. 2010;17(3):443–448.

5. Domingues-Montanari S, Hernandez-Guillamon M, Fernandez-Cadenas I, et al. ACE variants and risk of intracerebral hemorrhage recurrence in amyloid angiopathy [Internet]. Neurobiol. Aging 2011;32(3):551.e13-551.e22.Available from: http://dx.doi.org/10.1016/j.neurobiolaging.2010.01.019

6. Pera J, Slowik A, Dziedzic T, et al. Glutathione peroxidase 1 C593T polymorphism is associated with lobar intracerebral hemorrhage. Cerebrovasc. Dis. 2008;25(5):445–449.

7. Hallström B, Jönsson AC, Nerbrand C, et al. Stroke incidence and survival in the beginning of the 21st century in Southern Sweden: Comparisons with the late 20th century and projections into the future. Stroke 2008;39(1):10–15.

8. Deary IJ, Gow AJ, Pattie A, Starr JM. Cohort profile: The lothian birth cohorts of 1921 and 1936. Int. J. Epidemiol. 2012;41(6):1576–1584.

9. Pezzini A, Grassi M, Iacoviello L, et al. Serum cholesterol levels, HMG-CoA reductase inhibitors and the risk of intracerebral haemorrhage. The Multicenter Study on Cerebral

© 2019 Marini S et al. *JAMA Neurology*.

Haemorrhage in Italy (MUCH-Italy) [Internet]. J. Neurol. Neurosurg. Psychiatry 2016;87(9):924–929.Available from: http://jnnp.bmj.com/lookup/doi/10.1136/jnnp-2015-312736

10. Woo D, Falcone GJ, Devan WJ, et al. Meta-analysis of genome-wide association studies identifies 1q22 as a susceptibility locus for intracerebral hemorrhage. Am. J. Hum. Genet. 2014;94(4):511–521.

11. Anderson CD, Falcone GJ, Phuah CL, et al. Genetic variants in CETP increase risk of intracerebral hemorrhage. Ann. Neurol. 2016;80(5):730–740.

12. Walsh KB, Woo D, Sekar P, et al. Untreated Hypertension: A Powerful Risk Factor for Lobar and Nonlobar Intracerebral Hemorrhage in Whites, Blacks, and Hispanics. Circulation 2016;134(19):1444–1452.

13. Marini S, Morotti A, Ayres AM, et al. Sex differences in intracerebral hemorrhage expansion and mortality [Internet]. J. Neurol. Sci. 2017;379:112–116.Available from: http://dx.doi.org/10.1016/j.jns.2017.05.057

14. Woo D, Kissela BM, Khoury JC, et al. Hypercholesterolemia, HMG-CoA reductase inhibitors, and risk of intracerebral hemorrhage: a case-control study. [Internet]. Stroke. 2004;35(6):1360–4.Available from: http://www.ncbi.nlm.nih.gov/pubmed/15087556

15. Ariesen MJ, Claus SP, Rinkel GJE, Algra A. Risk factors for intracerebral hemorrhage in the general population: A systematic review. Stroke 2003;34(8):2060–2065.

16. Koch W, Ehrenhaft A, Griesser K, et al. TaqMan systems for genotyping of disease-related polymorphisms present in the gene encoding apolipoprotein E. Clin. Chem. Lab. Med. 2002;40(11):1123–1131.

17. Marini S, Lena UK, Crawford KM, et al. Comparison of Genetic and Self-Identified Ancestry in Modeling Intracerebral Hemorrhage Risk [Internet]. Front. Neurol. 2018;9:514.Available from: https://www.frontiersin.org/article/10.3389/fneur.2018.00514

18. Anderson C, Pettersson F, Clarke G, et al. Data quality control in genetic case-control association studies. Nat Protoc. 2010;5(9):1564–1573.

© 2019 Marini S et al. *JAMA Neurology*.

19. Howie BN, Donnelly P, Marchini J. A flexible and accurate genotype imputation method for the next generation of genome-wide association studies. PLoS Genet. 2009;5(6)

20. Price AL, Patterson NJ, Plenge RM, et al. Principal components analysis corrects for stratification in genome-wide association studies [Internet]. Nat. Genet. 2006;38(8):904–909.Available from: http://www.ncbi.nlm.nih.gov/pubmed/16862161%5Cnhttp://www.nature.com/doifinder/10.1038/ng1847

21. Purcell S, Neale B, Todd-Brown K, et al. PLINK: A tool set for whole-genome association and population-based linkage analyses. Am. J. Hum. Genet. 2007;81(3):559–575.

22. Biffi A. Genetic Variation and Neuroimaging Measures in Alzheimer Disease [Internet]. Arch. Neurol. 2010;67(6):677.Available from: http://archneur.jamanetwork.com/article.aspx?doi=10.1001/archneurol.2010.108

© 2019 Marini S et al. *JAMA Neurology*.
